# Targeting oral cancer with curcumin and its structural analogs: molecular mechanisms, pharmacokinetic optimization, and clinical translation

**DOI:** 10.1007/s12272-026-01635-8

**Published:** 2026-10-06

**Authors:** Shih-Chi Su, Chun-Wen Su, Chia-Yi Lee, Chiao-Wen Lin, Shun-Fa Yang

**Affiliations:** 1 Whole-Genome Research Core Laboratory of Human Diseases, Chang Gung Memorial Hospital, Keelung, Taiwan; 2 Department of Medical Biotechnology and Laboratory Science, College of Medicine, Chang Gung University, Taoyuan, Taiwan; 3 Institute of Medicine, Chung Shan Medical University, Taichung, 402 Taiwan; 4 Department of Medical Research, Chung Shan Medical University Hospital, Taichung, Taiwan; 5 Institute of Oral Sciences, Chung Shan Medical University, Taichung, Taiwan; 6 Department of Dentistry, Chung Shan Medical University Hospital, Taichung, Taiwan

**Keywords:** Curcumin, Oral cancer, Curcumin analogs, Pharmacokinetics, Molecular signaling pathways, Drug delivery systems

## Abstract

Curcumin is a naturally occurring polyphenol extracted from turmeric (*Curcuma longa*) and is known for its diverse biological functions, such as antioxidant, anti-inflammatory, anti-migratory, and anticancer activities. However, its use in clinical cancer treatment remains constrained due to challenges like limited absorption, rapid metabolic breakdown, a short half-life, and generally low bioavailability. To overcome these limitations, various curcumin analogs and formulation strategies have been developed to enhance its therapeutic efficacy. In oral cancer, a malignancy characterized by high recurrence rates, therapeutic resistance, and substantial treatment-related morbidity, novel multi-targeted and low-toxicity strategies remain urgently needed. Yet, the intersection between curcumin’s pharmacological constraints and the specific therapeutic vulnerabilities of oral cancer has not been critically synthesized. This review addresses this gap by integrating pharmacokinetic limitations, molecular mechanisms of action, and disease-specific signaling dependencies in oral cancer. We systematically examine evidence demonstrating that curcumin and its derivatives suppress proliferation, invasion, metastasis, angiogenesis, and inflammatory signaling through modulation of pathways such as nuclear factor-κB (NF-κB), phosphoinositide 3-kinase (PI3K)/Akt, mitogen-activated protein kinase (MAPK), and apoptosis-related cascades. Importantly, we critically evaluate next-generation curcumin analogs and advanced delivery systems that are designed to overcome bioavailability barriers and enhance tumor-specific efficacy. Collectively, the available evidence positions curcumin as an investigational multi-target scaffold, with its clinical translation in oral cancer dependent on rational formulation strategies and rigorous validation. Bridging mechanistic insights with pharmacokinetic optimization and well-designed clinical trials will be essential to define the therapeutic value of optimized curcumin-based adjunctive approaches.

## Introduction

Curcumin (diferuloylmethane) is the principal bioactive polyphenol derived from the rhizome of *Curcuma longa* (Shishodia et al. 2005; Kunnumakkara et al. 2017). It has attracted considerable interest as a multi-target anticancer compound because it can influence signaling networks, governing malignant cell survival, proliferation, invasion, inflammation, and stress responses (Goel and Aggarwal 2010; Kotha and Luthria 2019; Kuzminska et al. 2024). However, poor aqueous solubility, limited absorption, rapid metabolism, and short systemic persistence substantially restrict the exposure achievable with native curcumin (Anand et al. 2007; Dei Cas and Ghidoni 2019). These pharmacokinetic limitations are particularly important when evaluating whether the micromolar concentrations used in many preclinical oral cancer studies can be reproduced in clinically relevant tissues (Singh et al. 2023).

Several recent reviews have examined individual aspects of curcumin-based intervention in oral cancer (Table 1). A previous review article primarily summarized the molecular effects of curcumin reported in oral cancer models (Singh et al. 2023), whereas another focused predominantly on nanoformulation-based approaches (Mukherjee and Krishnan 2023). In addition, a subsequent review evaluated the therapeutic roles of curcumin and selected structural analogs in oral malignancies (Schiavoni et al. 2025). Unlike these prior reviews, the present review integrates molecular activity with pharmacologically achievable exposure and directly compares structural analogs and delivery platforms within an oral cancer-specific translational framework. Specifically, we link the major signaling vulnerabilities of oral cancer with the pharmacokinetic and pharmacodynamic constraints of curcumin. These vulnerabilities include epidermal growth factor receptor (EGFR), phosphoinositide 3-kinase (PI3K)/protein kinase B (Akt)/mammalian target of rapamycin (mTOR), signal transducer and activator of transcription 3 (STAT3), nuclear factor-κB (NF-κB), mitogen-activated protein kinase (MAPK), Wnt/β-catenin, epithelial-mesenchymal transition (EMT), cancer stemness, treatment resistance, and immune regulation. Native curcumin, structural analogs, and delivery platforms are further compared according to potency, chemical and metabolic stability, evidence of improved exposure, feasibility of local oral mucosal administration, combination-treatment potential, and stage of experimental or clinical validation. Through this translational framework, we aim to identify which curcumin-based strategies remain exploratory and which warrant further development as formulation-dependent adjunctive approaches for oral cancer.

**Table 1:** Comparison of the scope of the present review with selected previous reviews of curcumin in oral cancer

Review	Primary focus	Oral cancer signaling	Curcumin analogs	Delivery systems	PK/PD and exposure	Translational considerations
Singh et al. 2023	Molecular effects of curcumin in oral cancer	Individual signaling mechanisms	Limited coverage	Limited coverage	Not a major focus	Limited discussion
Mukherjee and Krishnan 2023	Curcumin nanoformulations in oral cancer	Partial coverage	Limited coverage	Principal focus	General discussion of bioavailability	Limited discussion
Schiavoni et al. 2025	Curcumin and selected analogs in OSCC	Included	Included	Included	Limited comparative discussion	Primarily preclinical therapeutic perspective
Present review (Su et al.)	Molecular, pharmacological, and translational aspects of curcumin-based strategies in oral cancer	Includes pathway cross-talk, resistance, immunity, and microbiome	Includes structure–activity relationships, stability, potency, and metabolic considerations	Includes systemic and local delivery approaches, feasibility, and scalability	Includes comparison of experimental activity with available exposure data	Includes pharmacokinetic, safety, validation-stage, and clinical-evidence considerations

*OSCC* oral squamous cell carcinoma, *PK/PD* pharmacokinetics/pharmacodynamics

## Literature search and selection strategy

A literature search was conducted to identify studies relevant to curcumin and its structural analogs in oral cancer. The search included the PubMed (National Center for Biotechnology Information) and Web of Science databases and covered publications available up to January, 2026. Search terms were used individually and in combination and included “curcumin,” “oral cancer,” “oral squamous cell carcinoma (OSCC),” “head and neck squamous cell carcinoma (HNSCC),” “curcumin analog,” “pharmacokinetics,” “bioavailability,” “drug delivery,” “nanoparticle,” “combination therapy,” “immunotherapy,” and “oral microbiome.” Priority was given to original studies directly involving oral cancer or OSCC, particularly those addressing molecular mechanisms, structural analogs, pharmacokinetic properties, delivery systems, treatment combinations, and clinical translation. Relevant reviews were used to provide background and to identify additional primary studies. Evidence from broader HNSCC or non-oral cancer models was included selectively when direct oral cancer evidence was unavailable or when it provided important pharmacokinetic, medicinal chemistry, or translational context.

## Chemical and pharmacological features of curcumin relevant to oral cancer

**Fig. 1:**
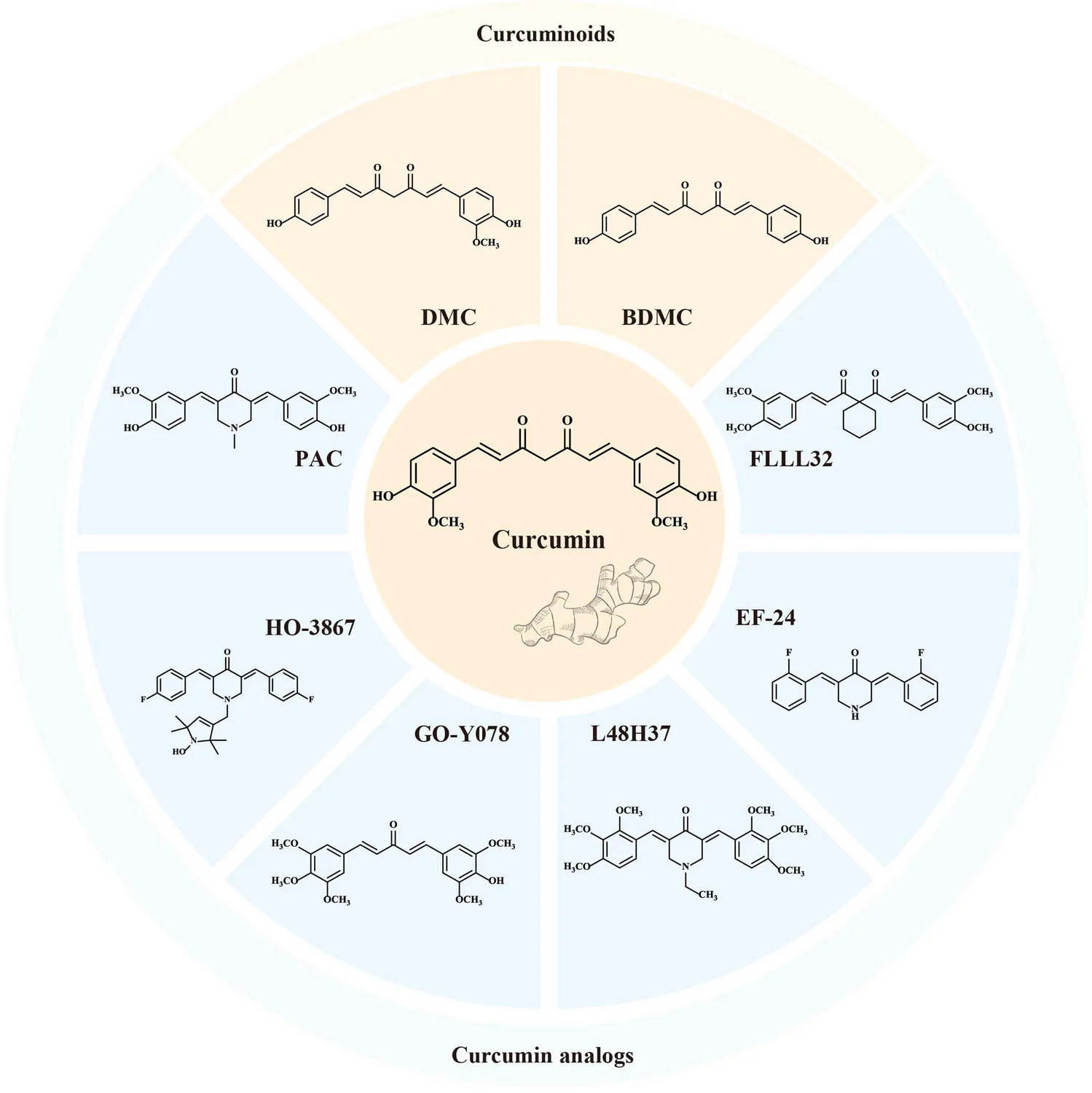
Schematic representation of curcumin and its representative natural derivatives and synthetic analogs. This figure summarizes the chemical structures of curcumin, its natural curcuminoids, including demethoxycurcumin (DMC) and bisdemethoxycurcumin (BDMC), as well as several representative synthetic curcumin analogs

The chemical structures of curcumin and its principal natural and synthetic analogs are shown in Fig. 1. Curcumin is a lipophilic diarylheptanoid containing two aromatic rings connected by a conjugated β-diketone linker (Mague et al. 2004; Dei Cas and Ghidoni 2019). These physicochemical features are associated with keto–enol tautomerism, chemical instability, poor aqueous solubility, and rapid metabolic transformation (Nelson et al. 2017; Tabanelli et al. 2021). Curcumin has been reported to influence numerous signaling proteins; however, its translational relevance in oral cancer depends less on the spectrum of its putative targets than on whether adequate concentrations can be achieved at the tumor site (Dhillon et al. 2008; Goel and Aggarwal 2010; Dei Cas and Ghidoni 2019). Accordingly, its broad molecular activity should be interpreted in relation to the limited systemic and tumor-site exposure achieved with native curcumin. These pharmacological constraints and their implications for oral cancer are examined in the following sections.

## Pharmacokinetic barriers and translational implications of curcumin

### Absorption, metabolism, and achievable exposure

Native curcumin exhibits poor oral bioavailability because of its limited aqueous solubility, low intestinal permeability, extensive metabolism, and rapid elimination (Tabanelli et al. 2021). Following oral administration, only a small proportion of unmetabolized curcumin enters the systemic circulation, whereas much of the administered dose remains unabsorbed or is eliminated through the gastrointestinal and biliary routes (Shoba et al. 1998; Tabanelli et al. 2021). Increasing the oral dose does not proportionally increase systemic exposure. In human pharmacokinetic studies, gram-level doses generally produced low and transient plasma concentrations, with circulating curcumin frequently declining to nearly undetectable levels within several hours (Shoba et al. 1998; Garcea et al. 2004; Dhillon et al. 2008).

Curcumin is also rapidly transformed in enterocytes and hepatocytes through glucuronidation, sulfation, and reductive metabolism. These reactions generate curcumin glucuronide, curcumin sulfate, and reduced metabolites such as dihydrocurcumin, tetrahydrocurcumin, and hexahydrocurcumin (Heger et al. 2014; Nelson et al. 2017; Dei Cas and Ghidoni 2019). Although some metabolites retain antioxidant, anti-inflammatory, or anticancer activities, their potency and molecular effects may differ from those of the parent compound (Prasad et al. 2014; Hatamipour et al. 2018). Consequently, biological effects observed after oral administration cannot necessarily be attributed exclusively to unmetabolized curcumin.

These pharmacokinetic properties create an important discrepancy between experimental activity and clinically achievable exposure. Many oral cancer studies report growth inhibition, apoptosis, or suppression of invasive signaling following exposure to micromolar concentrations of curcumin (Zhen et al. 2014; Liu et al. 2021a, b; Singh et al. 2023). In contrast, systemic exposure to unmetabolized curcumin after oral administration is generally low and short-lived, and typically remains below the concentrations used in cell-based experiments (Shoba et al. 1998; Garcea et al. 2004; Dhillon et al. 2008). Therefore, mechanistic findings obtained at relatively high concentrations should not automatically be interpreted as evidence that the same targets can be modulated in patients. In addition, the broad changes observed at high concentrations may reflect simultaneous perturbation of multiple signaling and cellular stress pathways rather than selective engagement of an individual molecular target. Evaluation of curcumin-based strategies should therefore include pharmacokinetic-pharmacodynamic relationships, measurement of free rather than total curcumin, verification of tumor or oral mucosal exposure, and comparison of effective tissue concentrations with those used in experimental models.

Clinical translation has also been constrained by heterogeneity in clinical development (Hegde et al. 2023). Early studies evaluated markedly different preparations and treatment regimens. These included standardized *Curcuma* extract containing 36–180 mg of curcumin daily, native curcumin at 8 g/day, theracurmin at 200–400 mg/day with chemotherapy, and weekly intravenous liposomal curcumin at 100–300 mg/m^2^ (Sharma et al. 2001; Dhillon et al. 2008; Kanai et al. 2013; Greil et al. 2018). These predominantly early-phase, single-arm studies enrolled small cohorts across different malignancies and used variable pharmacokinetic, biomarker, and efficacy endpoints, thereby providing limited cross-study comparison and exposure–response relationship. Although some studies measured plasma curcumin or peripheral-blood biomarkers, direct verification of intratumoral exposure and target engagement was uncommon, and biomarker-guided patient selection was generally not incorporated (Sharma et al. 2001; Dhillon et al. 2008; Kanai et al. 2013; Greil et al. 2018). Consequently, no standardized formulation, dosing optimization, or molecularly defined patient population has yet demonstrated reproducible anticancer properties. Importantly, these pharmacokinetic and early clinical observations were derived predominantly from non-oral malignancies and therefore provide general information on curcumin disposition rather than direct evidence of drug exposure or therapeutic activity in OSCC.

In addition, the accessibility of oral tumors creates an opportunity for local delivery approaches that may partly circumvent intestinal absorption and first-pass metabolism. Yet, evidence of increased formulation stability or plasma exposure alone is insufficient to establish therapeutic relevance. Future studies should directly measure local retention, mucosal penetration, intratumoral concentration, duration of exposure, and biological target modulation. These considerations provide the rationale for developing metabolically stable structural analogs and oral cavity-directed delivery systems, which are discussed in the following sections.

### Rationale for structural and formulation optimization

The limited exposure achieved with native curcumin has motivated two complementary development strategies (Tabanelli et al. 2021; Hegde et al. 2023). One approach involves structural modification of the curcumin scaffold to improve chemical stability and metabolic resistance and, in some cases, increase potency or target selectivity (Chainoglou and Hadjipavlou-Litina 2019; Bhatt et al. 2021). The second uses formulation-based approaches to enhance solubility, protect curcumin from degradation, prolong local or systemic retention, and facilitate tumor-directed delivery (Ban et al. 2020; Sideek et al. 2022; Mukherjee and Krishnan 2023; Rosa et al. 2023). These strategies are not equivalent: structural analogs create new chemical entities with potentially distinct pharmacological and toxicological properties, whereas delivery systems primarily seek to improve the disposition of curcumin without fundamentally altering its molecular structure. Their translational value therefore depends on different criteria, including pharmacokinetic improvement, reproducibility of drug exposure, safety, manufacturability, and evidence of biological target engagement. The medicinal chemistry of curcumin analogs and the comparative performance of delivery platforms are critically evaluated in Sects. "Therapeutic evaluation of curcumin analogs in oral cancer" and "Curcumin delivery platforms for oral cancer", respectively.

## Oral cancer

### Overview of oral cancer

Oral cancer comprises a heterogeneous group of malignancies arising within the oral cavity, of which OSCC accounts for approximately 90% of cases (Jagadeesan et al. 2024; Siegel et al. 2026). Tobacco smoking, alcohol consumption, and betel quid chewing are among the principal preventable risk factors, although their relative contributions vary geographically and may interact with genetic susceptibility, chronic mucosal injury, and poor oral hygiene (Zini et al. 2010; Jagadeesan et al. 2024). Among these established risks, betel quid exposure is particularly relevant in South and Southeast Asia and has been associated with distinct genomic alterations and gene-expression programs in OSCC (Su et al. 2025).

Although early-stage OSCC may be effectively managed by surgery, radiotherapy, or their combination, locally advanced and recurrent disease remains difficult to control because of invasive growth, cervical lymph-node metastasis, treatment resistance, and substantial functional morbidity (Johnson et al. 2020). Systemic treatment may improve disease control in selected patients, but its effectiveness is frequently constrained by toxicity, heterogeneous treatment responses, and the emergence of resistant tumor-cell populations (Bhat et al. 2021). These limitations have encouraged the development of adjunctive approaches capable of modulating multiple oncogenic and resistance-associated pathways. In this context, curcumin-based interventions are of interest not simply because of their broad molecular activity, but because their potential value in OSCC depends on whether sufficient exposure, potency, and target modulation can be achieved without increasing treatment-related toxicity.

### Molecular vulnerabilities associated with oral cancer

Due to the heterogeneous nature of disease etiology, OSCC is driven by interconnected oncogenic pathways. EGFR-associated signaling activates the RAS-RAF-ERK and PI3K/Akt/mTOR cascades, while STAT3, NF-κB, and Wnt/β-catenin further support tumor-cell proliferation, survival, inflammation, and treatment resistance (Karunakaran and Muniyan 2020; Georgaki et al. 2021; Dey et al. 2022). Extensive cross-talk and functional redundancy among these pathways may limit the durability of single-target inhibition and provide a rationale for evaluating multi-target interventions. However, this network complexity also reflects that changes in individual signaling molecules should not automatically be interpreted as evidence of direct target engagement. These pathways interact at several levels rather than functioning as independent signaling modules, allowing one branch to maintain proliferative or survival signaling when the other is suppressed. Akt-dependent regulation of glycogen synthase kinase−3β (GSK-3β) can also promote β-catenin accumulation, thereby connecting PI3K/Akt activity with Wnt-dependent transcription, migration, and invasion in OSCC (Che et al. 2023). In addition, ERK1/2 has been shown to modulate STAT3 phosphorylation and cell growth in OSCC cells, while NF-κB-dependent interleukin-6 (IL-6) production can sustain autocrine or paracrine STAT3 activation in head and neck cancer (Squarize et al. 2006; Gkouveris et al. 2014). These convergent interactions may support proliferation, stemness, inflammation, and treatment resistance despite inhibition of an individual pathway. Accordingly, curcumin-induced changes across several signaling proteins should be interpreted as modulation of an integrated signaling network rather than independent inhibition of multiple unrelated targets.

In addition to entwined oncogenic pathways, phenotypic plasticity further contributes to OSCC progression. EMT promotes invasion and metastasis and is closely linked to cancer stemness and resistance to chemotherapy and radiotherapy (Jayanthi et al. 2020). The integrin/focal adhesion kinase (FAK)/Src/ERK/β-catenin axis, for example, has been implicated in the regulation of oral cancer stemness and chemoresistance (Jang et al. 2022). The tumor microenvironment—including cancer-associated fibroblasts, endothelial cells, immune populations, and extracellular-matrix components—also supports tumor growth and treatment resistance, while hypoxia and oxidative stress modify these interactions (Kujan et al. 2017; Li et al. 2024a, b).

### Oral microbiome and curcumin-based modulation

Oral microbial dysbiosis may contribute to OSCC through chronic inflammation, altered mucosal immunity, and microbial carcinogen metabolism (La Rosa et al. 2020; Stasiewicz and Karpinski 2022; Li et al. 2023). Oral microorganisms can convert ethanol into acetaldehyde, and microbial samples collected from patients with OSCC or malignant oral mucosal disorders have produced potentially carcinogenic acetaldehyde concentrations following ethanol exposure (Gainza-Cirauqui et al. 2013; Marttila et al. 2013). The oral microbiome may also influence the response of OSCC treatment. Baseline microbial profiles have been associated with sensitivity to docetaxel–cisplatin–5-fluorouracil induction chemotherapy, whereas chemoradiotherapy has been linked to longitudinal changes in salivary microbial diversity and community composition (Rui et al. 2021; Medeiros et al. 2023). These findings support the oral microbiome as a potential biomarker with prognostic value, although causal relationships remain incompletely established.

Curcumin may affect this tumor–microbiome interface through both antimicrobial and host-directed activities. It inhibited the growth of *Porphyromonas gingivalis*, *Prevotella intermedia*, *Fusobacterium nucleatum*, and *Treponema denticola* and suppressed formation of biofilms (Izui et al. 2016). Curcumin also reduced IL-6, IL-1β, and tumor necrosis factor-α (TNF-α) expression and limited the entry and cytotoxicity of *Porphyromonas gingivalis* outer-membrane vesicles in human gingival epithelial cells (Izui et al. 2021). In an OSCC model, combining curcumin with *Lactobacillus rhamnosus* GG cell-free supernatant enhanced apoptosis in SCC-9 cells compared with either treatment alone (Udompatanakorn and Ratthawongjirakul 2024). However, direct evidence that curcumin reshapes oral microbial communities, reduces microbial carcinogen production, restores mucosal antitumor immunity, or improves treatment response in patients with OSCC is currently lacking. Future integration of longitudinal oral microbial profiling with measurements of microbial metabolites, mucosal immunity, and clinical outcomes could be a useful map of curcumin-mediated oral microbiome modulation in OSCC treatment.

## Curcumin in oral cancer

### Tumor-suppressive mechanisms of curcumin in oral cancer

**Fig. 2:**
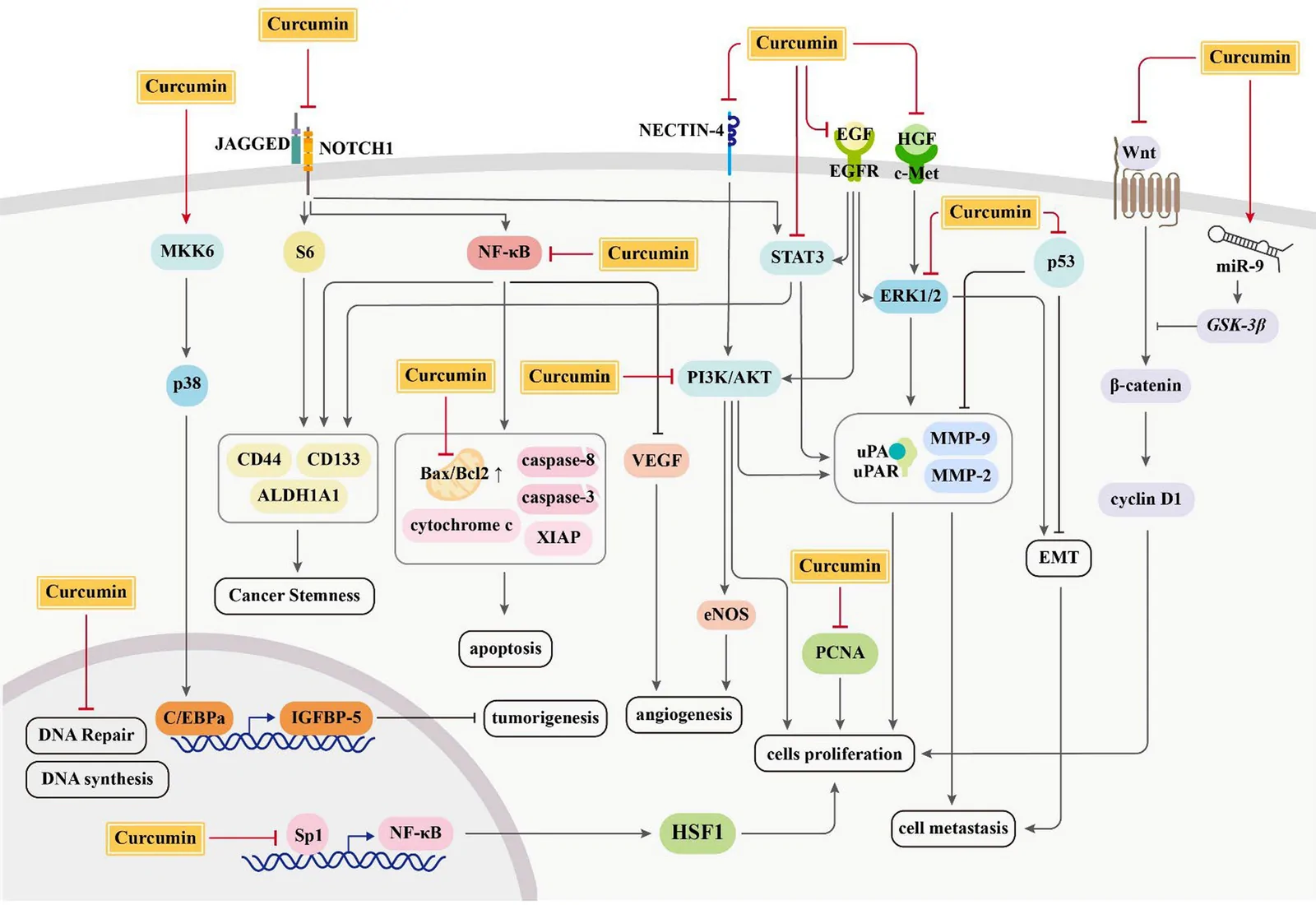
Schematic representation of the molecular signaling pathways modulated by curcumin in oral cancer cells. Schematic overview of the pleiotropic regulatory effects of curcumin on cancer-associated signaling pathways involved in cancer stemness, apoptosis, proliferation, angiogenesis, epithelial–mesenchymal transition (EMT), and metastasis. Curcumin has been reported to modulate multiple membrane-associated receptors and downstream signaling cascades, including NOTCH1, EGFR, c-Met, Wnt/β-catenin, STAT3, NF-κB, PI3K/Akt, ERK1/2, and p38. Through these pathways, curcumin regulates the expression and activity of key molecular mediators associated with cancer stemness (CD44, CD133, ALDH1A1), apoptosis (Bax/Bcl-2, cytochrome c, caspases, and XIAP), angiogenesis (VEGF and eNOS), cell proliferation (PCNA and cyclin D1), and metastasis-related processes (uPA/uPAR and MMP-2/9). *ALDH*1*A*1 aldehyde dehydrogenase 1 family member A1, *EGFR* epidermal growth factor receptor, *EMT* epithelial–mesenchymal transition, *eNOS* endothelial nitric oxide synthase, ERK extracellular signal-regulated kinase, *MMP* matrix metalloproteinase, *NF-κB* nuclear factor-κB, *PCNA* proliferating cell nuclear antigen, *PI*3*K* phosphoinositide 3-kinase, *STAT*3 signal transducer and activator of transcription 3, *uPA* urokinase-type plasminogen activator, *uPAR* uPA receptor, *VEGF* vascular endothelial growth factor, *XIAP* X-linked inhibitor of apoptosis protein

Curcumin exhibits a broad spectrum of anticancer activities in oral cancer through modulation of multiple molecular pathways (Zhao et al. 2019; Cheng et al. 2021; Singh et al. 2023). These include induction of apoptosis and autophagy, inhibition of cell proliferation, suppression of invasion and metastasis, regulation of angiogenesis, and modulation of inflammatory signaling (Sharma et al. 2006; Kim et al. 2012; Mishra et al. 2015; Maulina et al. 2019). Curcumin reduces oxidative stress, a key contributor to oral carcinogenesis, thereby suppressing tumor growth (Zheng et al. 2017). It also alters the tumor microenvironment and can influence the oral microbiota, both of which are increasingly recognized as important factors in oral cancer progression (Zlotogorski et al. 2013; Lopresti 2018; Stasiewicz and Karpinski 2022; Budi and Farhood 2023). The antitumor effects of curcumin on oral cancer reported in the literature are summarized in Table 2, highlighting the width of molecular targets and pathways affected. Curcumin exerts multiple antitumor effects in oral cancer by targeting diverse molecular pathways. It consistently suppresses key transcription factors and signaling proteins, including NF-κB, STAT3, specificity protein 1 (Sp1), and ERK1/2, leading to inhibition of cell proliferation and survival. Curcumin also modulates tumor suppressors and cell cycle regulators such as p53 and cyclin D1, induces apoptosis through caspase activation and Bax/Bcl-2 regulation, and promotes autophagy in certain models. In addition, it inhibits invasion, migration, and EMT by downregulating matrix metalloproteinases (MMPs), urokinase-type plasminogen activator (uPA)/uPA receptor (uPAR), Snail, Twist, and NECTIN-4 mediated signaling. Curcumin further impacts oxidative stress, angiogenesis, and DNA repair pathways, as evidenced by modulation of vascular endothelial growth factor (VEGF), endothelial nitric oxide synthase (eNOS), and base excision repair components. These effects have been observed across multiple oral cancer cell lines and in vivo models, demonstrating its pleiotropic anticancer activity (Fig. 2).

**Table 2:** Molecular mechanism of curcumin in oral cancer

Mechanism	Cell lines/in vivo	Testing dose	Reference
↓NF-κB, pS6, CD133, CD44, ALDH1A1, STAT3, NOTCH1	C57Bl/6 female mice	1 g/70 g body weight	Siddappa et al. 2017
↓PCNA, Bcl-2, SOCS1 e − 3, and STAT3	male albino rats	100 mg/kg	Gonçalves et al., 2015
↑C/EBPa, IGFBP-5, p38, MKK6	SAS cells	500 mg/kg	Chang et al. 2010
↓NF-κB, Sp1, p65 and HSF1	HSC3 and CAL33 cells	5‑20 μM	Liu et al. 2021a, b
↓growth and DNA synthesis	SCC-25 cells	0.1, 1, 10 μM	Elattar et al., 2000
↓MMP-2, MMP-9, uPA, uPAR, Akt, ERK1/2 and STAT3	SCC-25 cells	0, 10, 20, 40, 80 μM	Zhen et al. 2014
↑p53 ↓MMP-2, MMP-9, Snail, Twist, E-cadherin	SCC-25 cells	0, 2.5, 5, 10, 15, 30 μM	Lee et al. 2015
↓NFκB, ERK1/2, p-AKT, EMT	SCC-25 cells	2 μM	Dudás et al. 2013
↓HGF‑induced EMT and invasion, c‑Met, ERK, MMP-9	HSC‑4 and Ca9‑22 cells	15 μM	Ohnishi et al. 2020
↑miR-9, GSK-3b ↓Wnt, β-catenin, cyclin D1	SCC-9 cells	20, 40, 60 μM	Xiao et al. 2014
↓NECTIN-4PI3K-AKT, eNOS	H357 cells	0–30 μM	Chatterjee et al. 2021
↓short and long patch BER, PARylation	SCC-25 and H-357 cells	0–75 μM	Molla et al. 2020
↓Bax/Bcl2, cell viability	SCC-9 cells	5, 10, 20, 40, 80 μM	Udompatanakorn et al., 2024
↓cell viability	PECA-PJ15 cells	3, 3.75, 4.5, 5.25 μM	López-Jornet et al., 2011
↓cell viability, cell migration	PECA-PJ15 cells	3, 4.25, 5.50, 6.75 μM	Camacho-Alonso et al. 2013
↑cleaved caspase−3, cleaved caspase−8, cytochrome c ↓NF-κB, VEGF, MMP-9, BCL-2, XIAP	SAS cells	0–50 μM	Bhandarkar et al., 2007

*ERK extracellular signal‑regulated kinase*, *MMP* matrix metalloproteinase, *NF-κB* nuclear factor κ-light-chain enhancer of activated B cell, *PI3K* phosphoinositide 3-kinase, *STAT* signal transducer and activator of transcription, *XIAP* X-linked inhibitor of apoptosis protein

### Preclinical antitumor evidence and determinants of curcumin responsiveness in oral cancer

In an experimental study using 4-nitroquinoline−1-oxide (4-NQO)–induced oral carcinogenesis in mice, curcumin was administered daily at a concentration of 14.3 μg/mL (Siddappa et al. 2017). Curcumin treatment significantly reduced the number of carcinogenic lesions and oral tumors. Notably, 25 percent of animals receiving curcumin showed no evidence of oral cancer progression. In addition, curcumin administration decreased NF-κB expression and reduced the CD44⁺ cancer stem cell population (Siddappa et al. 2017). In a 4-NQO-induced rat oral cancer model, curcumin treatment at 100 mg/kg for three months significantly decreased the expression of proliferating cell nuclear antigen (PCNA), suppressor of cytokine signaling (SOCS)− 1 and SOCS-3, Bcl-2, and STAT3 (Goncalves Vde et al. 2015). Curcumin also reduced cellular atypia in this model. In a separate study, curcumin increased the nuclear expression of CCAAT/enhancer-binding protein alpha (C/EBPα) and insulin-like growth factor binding protein-5 (IGFBP-5) through activation of the p38 signaling pathway in murine oral cancer cells. Both proteins are known suppressors of head and neck carcinogenesis (Chang et al. 2010). In human oral cancer cell lines, curcumin markedly inhibited cell viability and colony-forming ability in HSC-3 and CAL-33 cells. This effect was accompanied by reduced expression of Sp1, p65, and heat shock factor 1 (HSF1), as well as decreased NF-κB activity through transcriptional suppression (Liu et al. 2021a, b). In SCC-25 human oral squamous cell carcinoma cells, curcumin in combination with cisplatin, a standard chemotherapeutic agent for oral cancer, induced a pronounced dose-dependent reduction in cell growth and proliferation compared with either treatment alone (Elattar and Virji 2000). Additional studies using SCC-25 cells demonstrated that curcumin inhibited cell proliferation and invasion, induced G2/M phase cell cycle arrest, and downregulated MMP-2, MMP-9, uPAR, phosphorylated EGFR, and downstream signaling molecules including Akt, ERK1/2, and STAT3 (Zhen et al. 2014). Curcumin treatment also regulated EMT markers such as Snail, Twist, and E-cadherin, and induced p53 expression in SCC-25 cells (Dudás et al. 2013; Lee et al. 2015). Furthermore, curcumin inhibited hepatocyte growth factor-induced vimentin expression, EMT, and cell motility in HSC-4 and Ca9-22 oral squamous cell carcinoma cells through suppression of ERK signaling, similar to the effects observed with c-Met blockade (Ohnishi et al. 2020). In SCC-9 cells, curcumin inhibited cell proliferation by upregulating microRNA-9 and suppressing the Wnt/β-catenin signaling pathway. This was associated with increased expression of GSK-3β, phosphorylated GSK-3β, and β-catenin, as well as reduced cyclin D1 levels (Xiao et al. 2014).

Curcumin has also been evaluated in combination with chemotherapy, molecularly targeted agents, poly(ADP-ribose) polymerase (PARP) inhibitors, and radiotherapy. The mechanistic rationale, strength of evidence, and translational relevance of these strategies are critically discussed in Sect. "Curcumin-based combination strategies in oral cancer".

Across the available oral cancer models, curcumin has shown relatively consistent antiproliferative, pro-apoptotic, and anti-invasive activity. However, the strength of these data remains uneven. Most mechanistic findings have been obtained from cultured oral cancer cells exposed to micromolar concentrations of curcumin, frequently without direct assessment of intracellular drug exposure or comparison with concentrations achievable in human plasma, tumor tissue, or the oral mucosa. Although chemically induced oral carcinogenesis models and tumor-bearing animals provide some in vivo observations, differences in species, dosing route, formulation, treatment duration, and experimental endpoints limit direct comparison among studies. The recurrent involvement of NF-κB, STAT3, PI3K/Akt, ERK, Wnt/β-catenin, and EMT-related regulators indicates convergence on interconnected survival and metastatic networks rather than inhibition of a single dominant target. Nevertheless, causal relationships are not always established because many studies rely mainly on changes in protein expression or phosphorylation. Combination studies involving chemotherapy or radiotherapy are encouraging but remain predominantly preclinical and have not yet demonstrated a consistently improved therapeutic index. Native curcumin should therefore be regarded as a preclinical multi-target adjunctive candidate whose translational value depends on standardized formulations and mechanistic validation in clinically relevant oral cancer models.

Moreover, resistance to curcumin itself remains underexplored in OSCC, but evidence suggests that several factors may influence treatment responsiveness. OSCC cell lines with different clinicopathological characteristics exhibit variable sensitivity to curcumin. In relatively insensitive cells, increasing intracellular copper enhanced reactive oxygen species generation, apoptosis, and growth inhibition, suggesting that cellular redox status may contribute to treatment response (Lee et al. 2016). Limited intracellular delivery may represent another barrier, as curcumin-loaded nanomicelles achieved greater cellular uptake and cytotoxicity than free curcumin in both parental and cisplatin-resistant oral cancer cells (Kumbar et al. 2022). More broadly, pathway redundancy, EMT and cancer stemness, and stress-adaptive autophagy may permit the persistence of a fraction of OSCC cells during treatment (Li et al. 2018; Jayanthi et al. 2020; Jang et al. 2022). Although these mechanisms have not been directly established as causes of acquired curcumin resistance, they provide testable candidates for future studies using curcumin-tolerant OSCC models.

## Therapeutic evaluation of curcumin analogs in oral cancer

### Medicinal chemistry rationale and structure–activity relationships

Curcumin contains two substituted aromatic rings connected by a flexible conjugated β-diketone linker. This scaffold is associated with keto–enol tautomerism and chemical instability, while the parent compound is also susceptible to rapid metabolism. Medicinal chemistry efforts have therefore focused on modifying the central linker, restricting molecular flexibility through cyclic or heterocyclic structures, and altering substituents on the terminal aromatic rings (Agrawal and Mishra 2010; Chainoglou and Hadjipavlou-Litina 2019). These modifications are intended to improve chemical stability, metabolic resistance, potency, or target selectivity. However, the anticipated benefits of structural modification should not be considered established unless improvements in solubility, pharmacokinetics, or therapeutic efficacy have been experimentally verified.

The curcumin analogs investigated in oral cancer represent structurally and pharmacologically heterogeneous compounds. Demethoxycurcumin (DMC) and bisdemethoxycurcumin (BDMC) retain the native β-diketone scaffold but lack one or both methoxy substituents. In contrast, GO-Y078 replaces the β-diketone moiety with an acyclic monocarbonyl linker, whereas EF-24, L48H37, HO-3867, and PAC contain cyclic 4-piperidone-based monocarbonyl scaffolds. FLLL32 retains two carbonyl groups but introduces a central spirocyclohexyl ring (Fig. 1). These structural modifications may alter physicochemical properties, cellular potency, and signaling responses. In OSCC models, FLLL32 and HO-3867 have been reported to induce apoptosis through p38 MAPK- and JNK1/2-associated signaling, respectively (Su et al. 2021; Chen et al. 2022). Yet, increased in vitro cytotoxicity alone does not establish improved bioavailability, metabolic stability, or tumor selectivity.

As summarized in Table 3, most oral cancer studies of these analogs have concentrated on cell viability, apoptosis, and signaling responses, with relatively limited assessment of aqueous solubility, metabolic half-life, systemic exposure, oral mucosal penetration, or intratumoral concentration. Direct comparisons with native curcumin are also inconsistent, and normal-cell toxicity or in vivo tolerability has not been uniformly examined. The following subsections therefore evaluate each analog according to its structural modification, medicinal chemistry rationale, comparative potency, selectivity, safety, and strength of pharmacokinetic evidence rather than considering anticancer activity alone.

**Table 3:** Medicinal chemistry and translational assessment of curcumin analogs evaluated in oral squamous cell carcinoma

Analog/scaffold	Key structural modification	Potential medicinal chemistry implication	Evidence in OSCC models	Translational status and main limitation	OSCC-specific references
FLLL32	Diketone analog in which the central methylene region is conformationally restricted by a spirocyclohexyl ring	Conformational restriction may reduce molecular flexibility and alter interactions with intracellular signaling proteins; the corresponding physicochemical advantage has not been established in OSCC	SCC-9 and HSC-3 cells; 0, 2, 4, and 8 μM. Reduced cell viability and induced G2/M arrest and caspase-dependent apoptosis involving HO-1 and p38 MAPK signaling	In vitro only; direct target binding and PK/PD relationships remain unverified	Su et al. 2021
EF-24	Symmetric fluorinated cyclic monocarbonyl analog based on a 4-piperidone scaffold	Replacement of the β-diketone with a cyclic monocarbonyl unit and fluorination may alter chemical stability, lipophilicity, and cellular potency; these advantages were not directly measured in the OSCC study	CAL-27 cells; 0.1–30 μM. Reduced cell viability and phosphorylation of MEK1, MAPK, and ERK, while increasing caspase−3 and caspase−9 activation	In vitro only; no oral cancer in vivo validation or exposure–response analysis is available	Lin et al. 2017
L48H37	Cyclic monocarbonyl analog containing an N-ethyl-4-piperidone core and two 3,4,5-trimethoxybenzylidene groups	The cyclic monocarbonyl scaffold and trimethoxy substitution may modify chemical stability, lipophilicity, and cellular uptake; these properties were not directly evaluated in OSCC	SCC-9 and HSC-3 cells; 0, 2.5, 5, 10, and 20 μM. Reduced viability and induced apoptosis through downregulation of cIAP1/XIAP, caspase activation, and JNK/p38 signaling	In vitro only; direct target engagement and pharmacokinetic improvement remain unverified	Su et al. 2024
GO-Y078	Asymmetric acyclic monocarbonyl analog containing a penta-1,4-dien-3-one linker and differently substituted aromatic rings	Removal of the β-diketone may reduce keto–enol tautomerism and alter chemical stability, but this proposed advantage has not been experimentally confirmed in OSCC	SCC-9 and HSC-3 cells; 0, 0.5, 1, 2, 4, and 8 μM. Inhibited proliferation and induced G2/M arrest and apoptosis involving SMAC/DIABLO, HO-1, AP-1, p38, and JNK1/2	In vitro only; no demonstrated pharmacokinetic or metabolic advantage in OSCC	Chien et al. 2022
HO-3867	Fluorinated cyclic monocarbonyl diarylidenyl-piperidone analog bearing an N-hydroxypyrroline-related substituent	The cyclic monocarbonyl scaffold, fluorinated aromatic rings, and N-substituent may alter redox behavior and cellular disposition; these properties were not measured in OSCC	SCC-9 and HSC-3 cells; 0, 2.5, 5, 10, and 20 μM. Suppressed cell growth and induced sub-G1 accumulation and caspase/PARP-dependent apoptosis through JNK1/2 signaling	In vitro only; no tissue-exposure or in vivo validation	Chen et al. 2022
DMC	Natural β-diketone curcuminoid lacking one methoxy substituent relative to curcumin	Reduced methoxy substitution may alter electronic and steric properties, but DMC retains the flexible β-diketone scaffold and its associated physicochemical liabilities	SCC-9 and HSC-3 cells; 0, 12.5, 25, and 50 μM. Inhibited proliferation and induced G2/M arrest and apoptosis through p38/JNK, HO-1, caspases, and downregulation of cIAP1/XIAP; also enhanced gefitinib activity	In vitro only, with gefitinib-combination evidence; no OSCC-specific pharmacokinetic or in vivo efficacy data are available	Chien et al. 2020
PAC	Cyclic monocarbonyl analog containing an N-methyl-4-piperidone core and two 4-hydroxy-3-methoxybenzylidene groups	Replacement of the β-diketone with a cyclic monocarbonyl scaffold may reduce tautomerism and alter cellular activity; OSCC-specific physicochemical improvement has not been demonstrated	Ca9-22 cells; 0, 1, 2.5, 5, and 10 μM. Induced apoptosis and autophagy and modulated oxidative-stress, NF-κB, MAPK, Wnt/β-catenin, and cell-cycle pathways. PAC also enhanced cisplatin-associated inhibition of proliferation and migration	In vitro only, with cisplatin-combination evidence; no oral cancer in vivo validation, dose-exposure analysis, or long-term safety assessment is available	Semlali et al. 2021; Semlali et al. 2023

Only OSCC-specific references are listed. Structural implications are presented as medicinal chemistry interpretations and should not be regarded as experimentally demonstrated improvements unless explicitly stated. *AP*−1 activator protein 1, *cIAP1* cellular inhibitor of apoptosis protein 1, *DMC* demethoxycurcumin, *ER* extracellular signal-regulated kinase, *HO*−1 heme oxygenase 1, *JNK* c-Jun N-terminal kinase, *MAPK* mitogen-activated protein kinase, *NF-κB*, nuclear factor kappa B, *OSCC* oral squamous cell carcinoma, *PAC* 3,5-bis(4-hydroxy-3-methoxybenzylidene)-N-methyl-4-piperidone, *PK/PD*, pharmacokinetic/pharmacodynamics, *XIAP* X-linked inhibitor of apoptosis protein

### Structurally Optimized Synthetic Curcumin Analogs

The synthetic curcumin analogs evaluated in OSCC differ in structural design and reported pathway engagement, but most have been assessed primarily through cell-based measures of proliferation, apoptosis, and signaling activity. Their OSCC-specific evidence is summarized below, with particular attention to whether the experimentally observed mechanisms support the intended medicinal chemistry rationale. FLLL32 and HO-3867 demonstrate that the mechanisms observed in oral cancer may differ from the target-directed rationale underlying their initial development. Both compounds were initially developed as STAT3-directed agents (Lin et al. 2010; Fossey et al. 2011; Rath et al. 2014), but their reported antitumor effects are associated with MAPK-mediated apoptotic responses in OSCC. FLLL32 reduced cell viability, induced G2/M arrest, and promoted caspase-dependent apoptosis involving p38 MAPK and heme oxygenase−1 (HO-1) signaling in SCC-9 and HSC-3 cells (Su et al. 2021). HO-3867 similarly induced sub-G1 accumulation and caspase- and PARP-dependent apoptosis, but its effects were associated primarily with JNK1/2 activation (Chen et al. 2022).

**Fig. 3:**
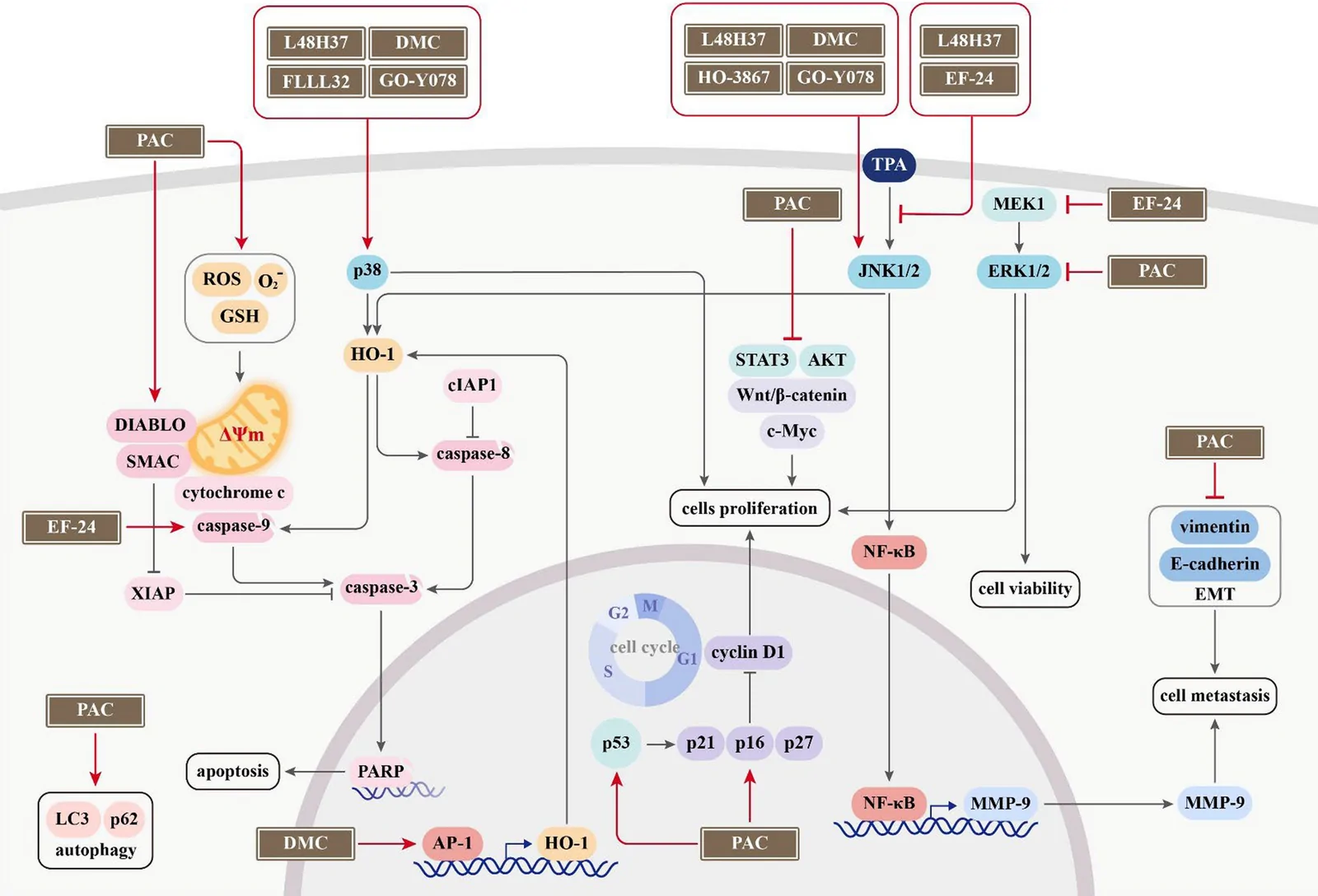
Schematic representation of the molecular signaling pathways regulated by curcumin analogs in oral cancer cells. Schematic representation of the reported molecular mechanisms by which representative curcumin analogs (PAC, EF-24, L48H37, FLLL32, DMC, GO-Y078, and HO-3867) modulate intracellular signaling pathways associated with apoptosis, autophagy, cell proliferation, cell cycle regulation, epithelial–mesenchymal transition (EMT), and metastasis. These curcumin-derived compounds have been shown to influence oxidative stress–related responses, mitochondrial dysfunction, and caspase-dependent apoptotic signaling, as well as MAPK-associated pathways, including p38, JNK, and ERK. In addition, curcumin analogs regulate oncogenic and survival-related signaling pathways, such as NF-κB, STAT3, Akt, and Wnt/β-catenin, thereby modulating the expression of downstream mediators involved in cell cycle progression (cyclin D1, p21, p16, and p27), apoptosis (caspases, PARP, and XIAP), and metastasis-related processes (MMP-9 and EMT-associated markers). *DMC* demethoxycurcumin, *EMT* epithelial–mesenchymal transition, *ERK* extracellular signal-regulated kinase, *JNK* c-Jun N-terminal kinase, *MAPK* mitogen-activated protein kinase, *MMP* matrix metalloproteinase, *NF-κB*, nuclear factor-κB, *PAC* 3,5-bis(4-hydroxy-3-methoxybenzylidene)-N-methyl-4-piperidone, *PARP* poly(ADP-ribose) polymerase, *STAT*3 signal transducer and activator of transcription 3, *XIAP* X-linked inhibitor of apoptosis protein

Other monocarbonyl analogs also converge on apoptosis- and stress-related pathways. EF-24 suppressed mitogen-activated protein kinase kinase 1 (MEK1)/MAPK/ERK phosphorylation and increased caspase−3 and caspase−9 activation in CAL-27 cells (Lin et al. 2017). L48H37 induced apoptosis in SCC-9 and HSC-3 cells through downregulation of cellular inhibitor of apoptosis protein 1 (cIAP1) and X-linked inhibitor of apoptosis protein (XIAP), activation of caspases, and engagement of JNK/p38 signaling (Su et al. 2024). GO-Y078 promoted G2/M arrest and apoptosis through SMAC/DIABLO and HO-1 upregulation, with HO-1 transcription linked to activator protein 1 (AP-1) activation downstream of p38 and JNK1/2 (Chien et al. 2022). PAC showed broader pathway modulation, including effects on NF-κB, MAPK, Wnt/β-catenin, oxidative-stress responses, apoptosis, and autophagy (Semlali et al. 2021), and also enhanced the antitumor activity of cisplatin in oral cancer cells (Semlali et al. 2023) (Fig. 3).

Collectively, these findings indicate that structural optimization of curcumin can generate compounds with substantial anti-neoplastic activity at low-to-moderate micromolar concentrations in OSCC cells. Of note, the reported mechanisms largely involve interconnected MAPK, apoptosis, and cellular-stress pathways rather than consistently validated direct molecular targets. Moreover, most studies remain limited to cell-based models and do not assess aqueous solubility, metabolic half-life, oral mucosal penetration, intratumoral exposure, or in vivo tolerability. The pharmacological advantage of these synthetic analogs over native curcumin therefore remains insufficiently established from a translational perspective, despite their encouraging in vitro activity.

### Natural curcuminoid analogs

Demethoxycurcumin (DMC) and bisdemethoxycurcumin (BDMC) are two natural curcuminoids that retain the β-diketone scaffold of curcumin but differ in methoxy substitution. In OSCC cell lines, DMC induced G2/M arrest and apoptosis through downregulation of cIAP1 and XIAP, induction of HO-1, activation of caspases, and engagement of p38 MAPK and JNK1/2 signaling (Chien et al. 2020). It also enhanced the antiproliferative effect of gefitinib (Chien et al. 2020). However, these findings remain limited to in vitro experiments, while normal-cell toxicity, pharmacokinetics, oral mucosal exposure, and in vivo efficacy have not been established. Although BDMC is reported as a naturally occurring phytochemical with antitumor activities against several types of malignancies (Ramezani et al. 2018), its direct OSCC evidence is currently insufficient.

### Comparative translational assessment

Collectively, the available OSCC studies indicate that structural modification of curcumin can generate compounds with substantial antiproliferative and pro-apoptotic activity across multiple oral cancer cell models. Synthetic analogs provide greater opportunities to optimize scaffold stability, potency, and pathway engagement, whereas natural curcuminoids retain close structural similarity to the parent compound. Among the candidates evaluated, PAC is particularly notable because it showed limited cytotoxicity toward normal human gingival cells and enhanced the activity of cisplatin (Semlali et al. 2021, 2023). DMC also strengthened the antiproliferative effect of gefitinib, supporting the potential use of curcumin analogs as adjunctive agents in targeted or conventional therapy (Chien et al. 2020).

FLLL32, L48H37, GO-Y078, and HO-3867 have likewise demonstrated consistent induction of apoptosis through MAPK- and stress-associated signaling in OSCC cells, identifying several candidate scaffolds for further optimization. Although direct pharmacokinetic and in vivo comparisons remain limited, these findings establish a useful preclinical foundation for candidate selection. Future studies incorporating matched comparisons with curcumin, oral mucosal or intratumoral exposure measurements, safety evaluation for normal tissues, and clinically relevant OSCC models will help determine which analogs are best suited for translational development. Collectively, the monocarbonyl, fluorinated, and conformationally restricted scaffolds represented by several curcumin analogs offer plausible advantages in chemical stability, metabolic resistance, and cellular potency. Although direct pharmacokinetic comparisons with native curcumin remain limited in OSCC models, these structural features provide a rational basis for prioritizing selected analogs for further exposure and efficacy studies. Current evidence therefore supports several candidate compounds for continued development, while additional pharmacokinetic validation is needed to determine their relative advantages (Table 3).

## Curcumin delivery platforms for oral cancer

### Oral cancer-specific demands for curcumin delivery

Curcumin-based formulations developed for OSCC should be tailored to the distinctive anatomical and physiological conditions of the oral cavity. An effective delivery system should improve curcumin solubility and chemical stability while ensuring adequate mucosal adhesion, controlled release, and penetration into tumor tissue. Its performance should therefore be assessed according to local retention, intratumoral drug concentration, duration of exposure, and pharmacodynamic target modulation rather than formulation characteristics or systemic exposure alone.

Oral cavity-directed formulations must balance adhesion to the mucosal surface with controlled drug release and tissue penetration. Thermosensitive hydrogels containing chitosan-coated, curcumin-loaded lipid-core nanocapsules have demonstrated strong mucoadhesion and prolonged oral mucosal retention (Ortega et al. 2023). Similarly, a curcumin-containing mucoadhesive emulgel provided sustained release over 24 h and permeation through a tissue-engineered oral mucosal model (Ferreira et al. 2020). These findings illustrate the potential value of localized and sustained delivery, although direct measurements of intratumoral curcumin concentrations and pharmacodynamic responses remain limited.

Safety and practical feasibility are equally important. Delivery platforms should preferentially increase drug exposure in malignant tissue without damaging adjacent normal oral mucosa; for example, chitosan-coated curcumin nanoparticles showed lower cytotoxicity toward normal cells than free curcumin in vitro (Mazzarino et al. 2015). Candidate formulations should also be assessed for batch reproducibility, storage stability, ease of administration, manufacturing scalability, and compatibility with chemotherapy or radiotherapy. Thus, ideal platforms for oral cancer should provide improved local exposure and therapeutic selectivity while remaining sufficiently stable, safe, and practical for clinical use (Table 4).

**Table 4:** Comparative translational assessment of curcumin delivery platforms investigated in oral cancer

Delivery platform/formulation	Representative oral cancer evidence	Key delivery advantages	Main limitations and translational considerations	Translational status/references
Micro- and nanoemulsions	Microemulsions suppressed OSCC-4/OSCC-25 cells, particularly with ultrasound. Nanoemulsions inhibited HSC-3 proliferation and modulated PI3K/Akt/mTOR and miR-199a	Improve dispersion and apparent solubility; compatible with ultrasound. A murine formulation study reported >40-fold higher Cmax than curcumin suspension	No prolonged mucosal retention or active targeting was shown. The PK study was not performed in an oral cancer model; surfactant tolerability and droplet stability require validation	In vitro OSCC; pharmacokinetic improvement demonstrated in a non-OSCC animal model. Lin et al. 2012; Zhongfa et al. 2012; Liu et al. 2023
Nanoliposomes	Curcumin/Dorema aucheri nanoliposomes induced apoptosis and reduced EGFR expression in OCC-02 cells	Protect lipophilic curcumin from degradation and permit sustained release or co-encapsulation	No PK, intratumoral exposure, mucosal retention, or active targeting data were reported. The specific contribution of curcumin is confounded by co-encapsulation; leakage and phospholipid oxidation are additional concerns	In vitro only. Azizi et al. 2023
Inorganic nanoparticles	Silica-encapsulated curcumin enhanced phototoxicity and NF-kB suppression; curcumin-mediated ZnO nanoparticles induced apoptosis-related responses in KB cells	High surface area and potential integration with photoactivated or multifunctional treatment	No controlled-release, PK, mucosal retention, or tumor-targeting evidence was provided. Particle persistence, material-associated toxicity, and long-term oral-tissue safety require assessment	In vitro only. Singh et al. 2014; Tayyeb et al., 2024
Chitosan-coated polymeric nanoparticles	Reduced SCC-9 viability and showed lower cytotoxicity toward normal cells than free curcumin	Combine encapsulation with a biocompatible, potentially mucoadhesive coating and possible normal-tissue sparing	Mucosal retention, penetration, PK, and intratumoral exposure were not directly measured. Polymer properties, coating uniformity, particle size, and storage stability require standardization	In vitro only; local-delivery potential. Mazzarino et al. 2015
Niosomes/nano-niosomes	Inhibited KB cells and reduced severe dysplasia in a rat oral carcinogenesis model	Provide vesicular encapsulation and slow release; topical administration is feasible and material costs may be lower than for liposomes	Tissue curcumin concentrations and PK-PD relationships were not measured. Surfactant safety, vesicle stability, sterilization, and scale-up reproducibility remain important	In vitro and rat in vivo validation. Fazli et al. 2022
Nanomicelles	Increased curcumin uptake, cytotoxicity, and apoptosis in parental and cisplatin-resistant KB cells	Efficiently solubilize hydrophobic curcumin and enhance intracellular delivery; potentially useful in resistant disease	No systemic PK, oral tumor exposure, mucosal retention, or targeting data were reported. Dilution-dependent instability, premature release, and polymer/surfactant safety remain concerns	In vitro only, including cisplatin-resistant cells. Kumbar et al. 2022
Thermosensitive mucoadhesive hydrogel with lipid-core nanocapsules	Demonstrated strong mucoadhesion, prolonged oral mucosal retention, and reduced SCC-25 viability	Combines in situ gelation, carrier-mediated protection, and sustained local release; provides the clearest evidence of prolonged mucosal retention	Mucosal penetration, intratumoral concentration, in vivo efficacy, local irritation, sterility, storage stability, and patient acceptability remain to be established	Formulation and in vitro evidence; oral mucosal retention demonstrated. Ortega et al. 2023
Mucoadhesive emulgel	Showed CAL27 cytotoxicity, sustained release, and permeation through tissue-engineered oral mucosa	Semisolid and locally applicable; combines solubilization, controlled release, and mucosal permeation and may be simpler than multifunctional nanocarriers	Comparative retention, tumor selectivity, in vivo exposure, salivary washout, taste, dosing frequency, local irritation, and long-term stability require evaluation	Preformulation and in vitro oral-mucosal evidence. Ferreira et al. 2020
Co-delivery platforms	Curcumin/gefitinib nanoparticles suppressed SAS xenografts. Curcumin/cisplatin niosomes and curcumin/5-fluorouracil nanohybrids enhanced activity in squamous carcinoma cells	Coordinate intracellular exposure, maintain a selected drug ratio, and may enhance sensitization or overcome resistance	No local retention or active targeting was demonstrated, and comparative PK is lacking. Loading efficiency, ratio drift, differential release, combination toxicity, and manufacturing reproducibility require control	In vitro evidence and one xenograft study; combination-treatment evidence. Lai et al. 2019; Srivastava et al. 2018; Saberian et al. 2025

Oral cancer and oral mucosal evidence is prioritized. The murine nanoemulsion pharmacokinetic study was not conducted in an oral cancer model, and active ligand- or receptor-directed targeting was not demonstrated in the cited studies. *Cmax* maximum plasma concentration, *EGFR* epidermal growth factor receptor, *miR* microRNA, *NF-kB*, nuclear factor kappa B, *OSCC* oral squamous cell carcinoma, *PI3K* phosphoinositide 3-kinase, *PK-PD* pharmacokinetic-pharmacodynamic, *ZnO*, zinc oxide

### Exposure-enhancing nanocarriers

Nanoemulsions, liposomes, polymeric or inorganic nanoparticles, micelles, and niosomes have been investigated to improve curcumin dispersion, protect it from degradation, and increase cellular or systemic exposure. More broadly, a systematic review of oral curcumin delivery systems found that nanocarrier-based formulations generally improved curcumin bioavailability, although most supporting evidence was derived from animal rather than clinical studies (Pan-On et al. 2022). Lipid-based emulsions provide particularly clear pharmacokinetic evidence: in a non-OSCC murine pharmacokinetic study, a curcumin nanoemulsion produced a more than 40-fold increase in maximum plasma concentration compared with a curcumin suspension (Zhongfa et al. 2012). On the other hand, oral cancer-specific studies have primarily evaluated cellular responses to micro- or nanoemulsion formulations rather than pharmacokinetic exposure. In oral cancer models, curcumin microemulsions suppressed OSCC cell growth, particularly when combined with ultrasound-mediated delivery (Lin et al. 2012), while nanoemulsions inhibited proliferation through modulation of PI3K/Akt/mTOR signaling and miR-199a expression (Liu et al. 2023). Nevertheless, the pharmacokinetic improvement reported in mice has not yet been directly linked to intratumoral exposure or efficacy in OSCC models.

Liposomal, polymeric, and inorganic nanoparticles provide additional opportunities for sustained release and modification of carrier composition. In OCC-02 oral cancer cells, nanoliposomes co-encapsulating curcumin and *Dorema aucheri* extract produced time-dependent cytotoxicity, apoptosis, and reduced EGFR expression, although the contribution of curcumin cannot be separated from that of the co-encapsulated extract (Azizi et al. 2023). Silica nanoparticle-encapsulated curcumin enhanced phototoxicity and suppression of NF-κB-regulated proteins (Singh et al. 2014), as chitosan-coated curcumin nanoparticles inhibited SCC-9 cell viability and exhibited lower cytotoxicity toward normal cells than free curcumin (Mazzarino et al. 2015). Curcumin-loaded nanomicelles also increased cellular uptake and apoptosis in both parental and cisplatin-resistant oral cancer cells (Kumbar et al. 2022).

Niosomal formulations have shown activity in oral cancer cells and reduced the development of severe dysplasia in a rat oral carcinogenesis model, providing preliminary support beyond cell-based experiments (Fazli et al. 2022). However, most exposure-enhancing nanocarriers have not been directly compared under matched experimental conditions. Their relative advantages must therefore be assessed according to drug-loading capacity, release kinetics, and safety for normal tissues. Although these systems improve curcumin delivery at the organism or cellular level, further pharmacokinetic-pharmacodynamic studies are required to establish whether they achieve sustained concentrations and target modulation within oral tumors.

### Local oral mucosal and mucoadhesive delivery

As exposure-enhancing nanocarriers primarily improve curcumin dispersion and cellular uptake, local mucosal systems are designed to retain the compound at the oral lesion and provide sustained release. Chitosan is particularly useful in this context because it can be incorporated as a surface coating or mucoadhesive component. Chitosan-coated curcumin nanoparticles inhibited SCC-9 cell viability while eliciting lower cytotoxicity toward normal cells than free curcumin (Mazzarino et al. 2015). In another approach, a thermosensitive hydrogel containing chitosan-coated, curcumin-loaded lipid-core nanocapsules demonstrated strong mucoadhesion, prolonged retention on the oral mucosa, and cytotoxic activity against OSCC cells (Ortega et al. 2023).

Mucoadhesive emulgels provide a semisolid alternative that combines drug solubilization with controlled local release. A curcumin-containing emulgel released approximately 70% of its incorporated curcumin over 24 h and demonstrated permeation through a tissue-engineered oral mucosal model (Ferreira et al. 2020). In vivo support for local administration has also been reported using a topical slow-release curcumin nano-niosome, which prevented the development of severe dysplasia in a rat oral carcinogenesis model (Fazli et al. 2022).

These findings support mucoadhesive delivery as a practical strategy for increasing local curcumin exposure while potentially reducing systemic drug requirements. Hydrogel–nanocapsule composites provide both carrier-mediated protection and prolonged mucosal retention, as emulgels may offer simpler administration and manufacturing. Nevertheless, active tumor-specific targeting has not been demonstrated, and comparative data on mucosal penetration, intratumoral concentration, local irritation, and dosing frequency remain limited. Future studies should therefore determine whether prolonged surface retention translates into adequate penetration and pharmacodynamic activity within oral tumors.

### Co-delivery platforms

Co-delivery platforms are designed to encapsulate curcumin together with a second therapeutic agent within the same carrier, thereby coordinating their release, cellular uptake, and relative exposure. This formulation-based strategy may be advantageous when treatment efficacy depends on maintaining a defined drug ratio or reducing differences in the pharmacokinetic behavior of individual agents. In oral cancer models, γ-polyglutamic acid-coated nanoparticles co-loaded with curcumin and gefitinib increased antitumor activity in SAS cells and xenograft-bearing mice compared with the corresponding free-drug combination (Lai et al. 2019). Moreover, niosomes co-encapsulating curcumin and cisplatin provided controlled release and showed the strongest activity at a curcumin-to-cisplatin ratio of 1:6 in OECM-1 cells (Saberian et al. 2025). Curcumin-5-fluorouracil nanoemulsions likewise improved cellular uptake of both agents and reduced their effective concentrations in SCC090 tongue cancer and SCC152 hypopharyngeal cancer cells (Srivastava et al. 2018). These findings suggest that carrier-based co-delivery can provide more consistent simultaneous exposure than separately administered drugs.

However, co-encapsulation also increases formulation complexity. Differences in drug solubility, loading efficiency, release kinetics, and chemical stability may make it difficult to preserve the intended drug ratio during storage and after administration. Additional components may also introduce carrier-related toxicity. Future studies should therefore compare co-loaded systems with both free-drug combinations and corresponding single-agent carriers while evaluating formulation stability, synchronized release, tissue exposure, and safety in normal oral mucosa.

### Comparative translational assessment

Curcumin delivery platforms provide distinct but complementary advantages in oral cancer. Nanoemulsions, liposomes, nanoparticles, micelles, and niosomes mainly improve solubility, stability, cellular uptake, and systemic exposure, whereas mucoadhesive hydrogels, nanocapsules, and emulgels are better suited to prolonged local retention and controlled delivery to oral lesions. Co-delivery systems may further enhance combination therapy by coordinating curcumin exposure with agents such as cisplatin, gefitinib, or 5-fluorouracil. Overall, local mucoadhesive formulations may be more feasible for localized disease, while multifunctional nanocarriers offer greater versatility for advanced or treatment-resistant OSCC.

From a translational perspective, anticancer effect of native curcumin is supported by limited in vivo oral carcinogenesis and combination-treatment studies, but its clinical development remains constrained by low and variable tumor exposure (Duarte et al. 2010; Chiang et al. 2014; Goncalves Vde et al. 2015; Siddappa et al. 2017). In contrast, the structural analogs evaluated in OSCC remain almost at the in vitro stage; although DMC and PAC have shown combination-treatment potential with gefitinib and cisplatin, respectively (Chien et al. 2020; Semlali et al. 2023). Delivery platforms also differ in their stage of development. Nanoemulsions have supporting pharmacokinetic evidence in non-OSCC models, whereas niosomes have undergone in vivo evaluation. Mucoadhesive hydrogels and emulgels demonstrate feasibility for localized oral delivery (Zhongfa et al. 2012; Ferreira et al. 2020; Fazli et al. 2022; Ortega et al. 2023) (Table 4). Nevertheless, no curcumin analog or delivery platform has yet achieved clinical validation in oral cancer. Local mucosal formulations may currently offer the most disease-specific route toward translation, but intratumoral exposure, safety, and clinical efficacy remain to be established.

## Curcumin-based combination strategies in oral cancer

### Combination with chemotherapy and radiotherapy

Curcumin-based combinations with cisplatin or radiotherapy have shown treatment-sensitizing effects in oral cancer models. Liposomal curcumin combined with cisplatin enhanced growth inhibition in CAL27 and UM-SCC1 cells and suppressed xenograft growth, potentially through inhibition of inhibitor of nuclear factor-κB kinase subunit β (IKKβ)/NF-κB signaling together with cisplatin-induced p53-associated senescence (Duarte et al. 2010). The curcumin analog PAC also strengthened cisplatin-induced apoptosis, oxidative stress, DNA damage, autophagy, and migration inhibition in Ca9-22 cells, suggesting possible dose-sparing activity (Semlali et al. 2023).

Curcumin has likewise enhanced the effects of irradiation in OSCC models. Combined treatment produced greater inhibition of viability and migration than either intervention alone in PE/CA-PJ15 cells, while curcumin improved tumor control in SAS/luc xenografts by suppressing radiation-induced NF-κB signaling (Lopez-Jornet et al. 2011; Camacho-Alonso et al. 2013; Chiang et al. 2014). These findings support further evaluation of curcumin as an adjunct for locally advanced or treatment-resistant OSCC. Nevertheless, the evidence remains preclinical, and future investigations should define optimal treatment sequence, intratumoral exposure, formal synergy, and effects on normal oral tissues before concluding that these combinations improve the therapeutic index.

### Combination with molecularly targeted and DNA-repair therapies

In cisplatin-resistant oral cancer cells, curcumin combined with cetuximab increased apoptosis and caspase activation while suppressing EGFR expression and MAPK signaling (Chen et al. 2018). DMC also enhanced the antiproliferative effect of gefitinib in OSCC cells, whereas nanoparticles co-loaded with curcumin and gefitinib improved apoptosis and xenograft control compared with the free-drug combination (Lai et al. 2019; Chien et al. 2020). These findings support further evaluation in EGFR-dependent or treatment-resistant OSCC, although predictive biomarkers, normal-tissue toxicity, and the contribution of curcumin beyond optimized EGFR inhibition remain insufficiently explored.

DNA-repair inhibition provides another complementary strategy. Curcumin combined with olaparib or talazoparib enhanced PARP trapping, disrupted base-excision repair, and promoted cell-cycle arrest and apoptosis in oral cancer cells (Chatterjee et al. 2022). In OSCC xenograft models, the curcumin–olaparib combination increased DNA damage, reduced PARylation, and suppressed tumor growth, whereas veliparib enhanced curcumin-mediated inhibition of NECTIN-4-associated angiogenic signaling (Molla et al. 2020; Chatterjee et al. 2021). However, these combinations remain preclinical, and their clinical development will require biomarker-guided selection of tumors with relevant DNA-repair vulnerabilities.

### Immunomodulatory effects and combination with immune checkpoint inhibitors

Evidence relevant to the immunomodulatory activity of curcumin currently derives from several distinct disease contexts. In broader HNSCC, immune checkpoint inhibitors, particularly pembrolizumab and nivolumab, are established treatments for recurrent or metastatic disease, providing a clinical rationale for investigating immunomodulatory combinations (Ferris et al. 2016; Burtness et al. 2019). In ex vivo HNSCC tissues, curcumin suppressed NF-κB activity, reduced the regulatory T cell (Treg)-attracting chemokine CCL22 (C–C motif chemokine ligand 22), and inhibited Treg recruitment, suggesting that it may reduce tumor-associated immunosuppression (Kotting et al. 2021).

OSCC-specific evidence is relatively limited. In a CAL27–macrophage coculture model, curcumin reprogrammed macrophage populations from an M2-like (anti-inflammatory) toward an M1-like phenotype (pro-inflammatory) through inhibition of monoamine oxidase A (MAO-A)/STAT6 signaling (Jiang et al. 2022). A recent in vitro study exhibited that curcumin treatment reduced programmed cell death protein 1 (PD-1) and programmed death-ligand 1 (PD-L1) levels in peripheral blood mononuclear cells obtained from patients with OSCC (Dash et al. 2025), although this observation does not establish reversal of T-cell exhaustion. Direct evidence that curcumin enhances the efficacy of anti-PD-1 or anti-PD-L1 therapy in oral cancer is currently lacking. Moreover, reduced checkpoint expression should not automatically be interpreted as improved antitumor immunity. Although PD-L1 may mediate immune evasion, its expression may also represent an adaptive response to interferon-γ signaling within a lymphocyte-infiltrated tumor microenvironment (Cho et al. 2011; Chen et al. 2012). Synergistic activity of curcumin with anti-PD-1 therapy has been demonstrated in hepatic cancer models (Guo et al. 2021); however, this non-oral cancer evidence should be regarded as hypothesis-generating rather than evidence of efficacy in OSCC. Future studies should therefore assess curcumin–checkpoint inhibitor combinations in immunocompetent OSCC models while examining tumor-infiltrating immune cells, cytokine responses, checkpoint expression, and immune-related toxicity.

Overall, the available evidence positions curcumin primarily as an investigational treatment-sensitizing adjunct, with clinical development dependent on standardized formulations, adequate tumor exposure, and biomarker-guided patient selection.

## Clinical evidence in oral and head and neck cancer

Although curcumin has undergone clinical evaluation in several malignancies, direct therapeutic evidence in oral cancer remains limited. More broadly, a recent critical analysis of clinical studies across malignant diseases found that improvements in curcumin bioavailability have not yet translated into convincing evidence of anticancer efficacy (Khosravi and Seifert 2024). A randomized phase I trial of the oromucosal formulation APG-157 in patients with oral cancer demonstrated tolerability, systemic absorption of curcumin-related compounds. Such treatment also modulated salivary inflammatory markers and tumor-associated microbial profiles, although the study was not designed to evaluate tumor response or survival (Basak et al. 2020). A follow-up phase II study is currently evaluating APG-157 as neoadjuvant or induction therapy in patients with newly diagnosed oral cavity or oropharyngeal HNSCC, but efficacy results have not yet been reported (NCT05312710). Evidence from oral leukoplakia represents a chemopreventive rather than established-cancer setting: a phase IIb trial reported a greater clinical response with curcumin than with placebo, whereas histological improvement was not significant (Kuriakose et al. 2016). Curcumin-containing formulations have also been evaluated for radiation-induced oral mucositis in patients with head and neck cancer, but these studies represent supportive-care applications rather than evidence of direct antitumor efficacy (Arun et al. 2020; Shah et al. 2020; Martins et al. 2023; Thomas et al. 2023). Thus, the available clinical literature spans oral cancer, broader HNSCC, oral premalignancy, and supportive-care settings and should not be interpreted as a single body of direct therapeutic evidence for OSCC.

## Conclusion and future directions

**Fig. 4:**
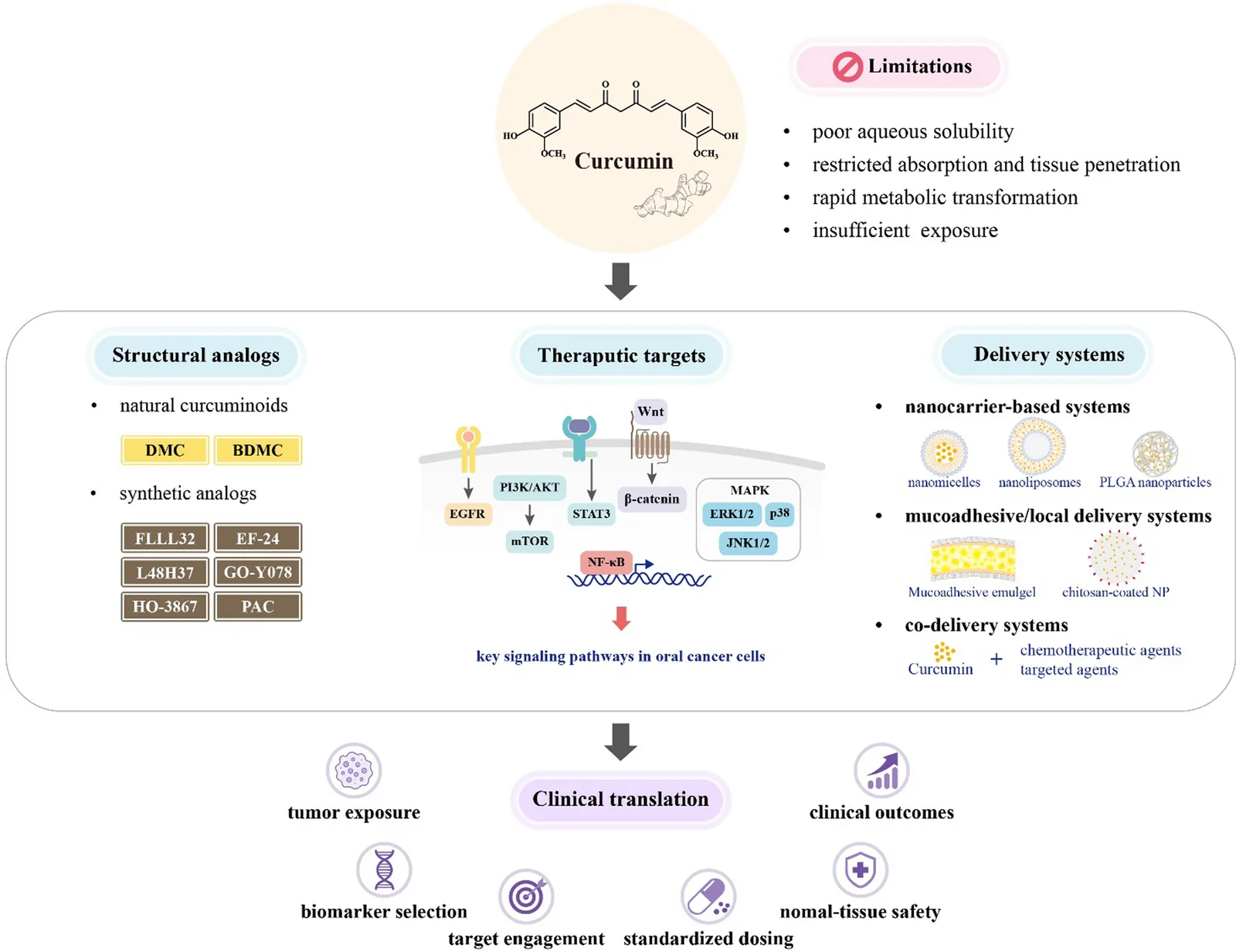
Integrated translational framework for curcumin-based strategies in oral cancer. Native curcumin is limited by poor aqueous solubility, restricted absorption and tissue penetration, rapid metabolic transformation, and low or transient systemic and tumor exposure. These pharmacokinetic constraints create a discrepancy between the concentrations used in many experimental studies and those likely to be achieved clinically. Structural analogs, exposure-enhancing nanocarriers, oral mucosal delivery systems, and co-delivery platforms represent complementary strategies for improving chemical stability, potency, local retention, tissue penetration, and combination-treatment exposure. Their therapeutic relevance depends on effective modulation of interconnected oral cancer vulnerabilities, including EGFR, PI3K/Akt/mTOR, STAT3, NF-κB, MAPK, Wnt/β-catenin, EMT, cancer stemness, immune regulation, and treatment resistance. Successful translation further requires demonstration of intratumoral exposure, pharmacodynamic target engagement, normal-tissue safety, standardized formulation and dosing, biomarker-guided patient selection, and clinically meaningful outcomes. *CBR* carbonyl reductase, *EGFR* epidermal growth factor receptor, *EMT* epithelial–mesenchymal transition, *MAPK* mitogen-activated protein kinase, *mTOR* mammalian target of rapamycin; *NF-κB* nuclear factor-κB, *PI3K* phosphoinositide 3-kinase, *STAT*3 signal transducer and activator of transcription 3, *SULT* sulfotransferase, *UGT* UDP-glucuronosyltransferase

Curcumin and its structural analogs exert broad antitumor effects in oral cancer models, including inhibition of proliferation, invasion, inflammatory signaling, and treatment resistance. However, their clinical value remains unproven because most evidence is preclinical, native curcumin achieves limited tumor exposure, and improved pharmacokinetics has not consistently been linked to target engagement or therapeutic benefit. Future development should prioritize analogs with improved stability, potency, selectivity, and tolerability, together with oral mucosal delivery systems that provide sustained local exposure and adequate tissue penetration. Both approaches require direct measurement of intratumoral concentrations and pharmacodynamic activity. Combination strategies involving chemotherapy, radiotherapy, EGFR inhibitors, PARP inhibitors, or immune checkpoint blockade should establish formal synergy, optimal treatment sequence, dose-sparing effects, and safety in normal oral tissues. Clinical trials should use standardized formulations, pharmacokinetically informed dosing, biomarker-guided patient selection, and adequately comparative designs. Clinical outcomes, such as tumor response, recurrence, survival, and treatment-related toxicity, should be prioritized to determine whether optimized curcumin-based strategies provide a reproducible therapeutic advantage in oral cancer. The integrated relationship among pharmacokinetic barriers, oral cancer-specific therapeutic vulnerabilities, optimization strategies, and the requirements for clinical translation is summarized in Fig. 4.

## Data Availability

No data were generated in the article.
